# Enhancing fine retinal vessel segmentation: Morphological reconstruction and double thresholds filtering strategy

**DOI:** 10.1371/journal.pone.0288792

**Published:** 2023-07-19

**Authors:** Hesham Abdushkour, Toufique A. Soomro, Ahmed Ali, Fayyaz Ali Jandan, Herbert Jelinek, Farida Memon, Faisal Althobiani, Saleh Mohammed Ghonaim, Muhammad Irfan

**Affiliations:** 1 Nautical Science Deptartment, Faculty of Maritime, King Abdul Aziz University, Jeddah, Saudia Arabia; 2 Department of Electronic Engineering, Quaid-e-Awam University of Engineering, Science and Technology Larkana Campus, Sukkur, Pakistan; 3 Eletrical Engineering Department, Sukkur IBA University, Sukkur, Pakistan; 4 Eletrical Engineering Department, Quaid-e-Awam University of Engineering, Science and Technology Larkana Campus, Sukkur, Pakistan; 5 Health Engineering Innovation Center and biotechnology Center, Khalifa University, Abu Dhabi, UAE; 6 Department of Electronic Engineering, Mehran University, Janshoro, Jamshoro, Pakistan; 7 Marine Engineering Department, Faculty of Maritime, King Abdul Aziz University, Jeddah, Saudia Arabia; 8 Electrical Engineering Department, College of Engineering, Najran University, Najran, Saudi Arabia; UNSW: University of New South Wales, AUSTRALIA

## Abstract

Eye diseases such as diabetic retinopathy are progressive with various changes in the retinal vessels, and it is difficult to analyze the disease for future treatment. There are many computerized algorithms implemented for retinal vessel segmentation, but the tiny vessels drop off, impacting the performance of the overall algorithms. This research work contains the new image processing techniques such as enhancement filters, coherence filters and binary thresholding techniques to handle the different color retinal fundus image problems to achieve a vessel image that is well-segmented, and the proposed algorithm has improved performance over existing work. Our developed technique incorporates morphological techniques to address the center light reflex issue. Additionally, to effectively resolve the problem of insufficient and varying contrast, our developed technique employs homomorphic methods and Wiener filtering. Coherent filters are used to address the coherence issue of the retina vessels, and then a double thresholding technique is applied with image reconstruction to achieve a correctly segmented vessel image. The results of our developed technique were evaluated using the STARE and DRIVE datasets and it achieves an accuracy of about 0.96 and a sensitivity of 0.81. The performance obtained from our proposed method proved the capability of the method which can be used by ophthalmology experts to diagnose ocular abnormalities and recommended for further treatment.

## 1 Introduction

Fundus images are just one source for ophthalmologists to analyze and diagnose different eye diseases. Therefore, digital color fundus images are used nowadays and digital image processing techniques are used to analyze different features of retinal images including abnormalities, optic papilla and retinal vessels. This computerized analysis greatly contributes to ophthalmologists in studying images for disease detection. This analysis helps to diagnose the severity of diseases such as glaucoma, blindness and macular edema [1–3]. Analysis of retinal vessel segmentation from retinal vessel network is mostly performed manually, but it is time consuming and yields errors for large patient databases for eye disease screening [4]. Computerized methods are implemented for accurate segmentation of retinal vessels, but there are different conditions of vessels such as some vessels are at the border of images, some contain pathologies and many of them are tiny vessels. Due to these vessel conditions, it becomes difficult for proper vessel segmentation [5, 6]. The computerized methods which require treating all these retinal vessel problems and giving accurate images of the vessels.

Retinal vessel segmentation is the process of identifying the blood vessels in the retina of the eye from fundus images. It plays a crucial role in the diagnosis and treatment of various retinal diseases, such as diabetic retinopathy, age-related macular degeneration, and glaucoma. Previous retinal vessel segmentation methods have been categorized into different techniques based on their underlying principles. Transform-based methods, also known as frequency-domain methods, process the images in the frequency domain by applying Fourier or wavelet transforms. These methods are efficient in detecting the large vessels but may struggle with detecting tiny vessels. Filtering-based methods use image filters, such as matched filters, Gabor filters, and vesselness filters, to enhance the blood vessels’ appearance in the image. These methods can detect vessels of different sizes but may be sensitive to noise and other image artifacts. Machine learning-based methods rely on the use of classifiers to differentiate between pixels that belong to blood vessels and those that do not. These methods require the availability of labeled data for training, and they can achieve high accuracy in vessel segmentation [7]. However, they may struggle with detecting small vessels due to the limited availability of training data. Recently, deep learning has become the most commonly used approach for retinal vessel segmentation. Deep learning models, such as convolutional neural networks (CNNs), have demonstrated superior performance in vessel segmentation compared to previous methods. However, these models may still struggle with accurately detecting tiny vessels due to the limited resolution of fundus images. Researchers continue to explore new techniques [8, 9] to overcome this challenge and improve the accuracy of retinal vessel segmentation.

Existing vessel segmentation methods face several challenges that limit their performance. One major issue is that the optic disc region’s brightness properties differ significantly from the rest of the image [10, 11]. The optic disc margin has a distinct brightness, making it challenging to locate vessels inside the disc. This often results in the edge of the optic disc appearing as a spurious vascular component in some retinal images. Another challenge is the presence of a mix of high-contrast multiple-pixel vessels of wide width and single-pixel wide low contrast vessels in retinal images [12]. These vessels may vary significantly in size and shape, and accurately detecting them using a single detector is difficult [13, 14]. Furthermore, current methods struggle to accurately segment thin or small vessels, which significantly affects their sensitivity. Detecting these tiny vessels is crucial for detecting early signs of diseases such as diabetic retinopathy and age-related macular degeneration. To address these challenges, this study focuses on systematically segregating the vessel segmentation problems, especially those related to tiny vessels. The aim is to develop more effective methods that can accurately detect vessels in the challenging optic disc region and address the difficulties posed by the mixture of vessel types and sizes in retinal images.

Over the past two decades, several researchers have implemented methods to solve retinal image segmentation problems, and retinal vessel segmentation methods are categorized into supervised and unsupervised methods. Supervised retinal vessel segmentation techniques are relied on pixel classification of vessels and non-vessels through a trained model. Supervised retinal segmentation methods required the database to train model for obtain images of segmented vessels. These methods are based on machine learning, deep learning as well as other AI approaches [15, 16]. Most classifiers such as Random Forest [17, 18], K Nearest Neighbors (KNN) [19], Support Vector Machine (SVM) [20, 21], Artificial Neural Networks (ANN) [22] and others are used to classify vessel images. Deep learning models gave good performance compared to other models, but training is time consuming and the model’s reliance on training samples with corresponding annotations [23, 24], which may not always be accessible, can pose a significant challenge, in particular for the segmentations of the retinal vessels.

Unsupervised retinal vessel segmentation methods are based on training data or require no prior information to segment the image. These methods are based on internal pixel tracking information or filtering schemes [25–27] such as vessel tracking, matching filtering, multi-scale, mathematical modeling based on morphological operations as well as machine learning as well. Many researchers have discussed these methods in detail because unsupervised methods have the advantage of higher speed and they take less computation time, but they still need a lot of improvement because their performance, in especially sensitivity, are measured at a low level compared to supervised methods. There are other issues in unsupervised methods such as hyperparameter tuning, large memory requirements, and low-resolution image enhancements that are attributed due to image acquisitions such as camera type, noise as well as patient movement. In this research work, we will implement the unsupervised method which solves all the problems mentioned above.

The proposed unsupervised method solves the first problems by using the pixel-based image processing tactics and filters without obtaining prior information to tune the parameters. The proposed method is provided the solution of the research questions of this research work which clearly define the contribution of this research work. The first question of this research work is how the proposed methods solve image acquisitions with reduce noise and maintain image quality?. The second question is: In what way does the proposed method effectively mitigate low-varying contrast issues?. The third question, how do the proposed methods ensure vessel coherency during the initial phase of vessel segmentation?. The fourth question is: how do the proposed methods provide well-segmented images of vessels without dropping tiny vessels?. The last question is to know what are the main contributions of the proposed methods?. The answer to all of the proposed questions is listed below:

1. The proposed method solves the problem of noise removal by using the new tactics of conversion of red-green-blue (RGB) based on inverted images according to the mask of the images, since it kept the boundary pixels. We used morphological operations to maintain the light reflex center as well. These two steps maintain the image quality for further process.
2. After the acquisition process, the image still suffered from insufficient and varying contrast issues, which are crucial to resolve in order to obtain a grayscale image with good contrast. We used the homomorphic technique to solve the contrast problem, then after adjusting the contrast using Wiener filtering and selecting the best contrast image from RGB by measuring the noise ratio and contrast ratio of the images.
3. After improving the low-varying contrast and getting the well contrasted image, we used the combination of two contrast normalization filtering, one is a dimensional Laplacian Gaussian filter (LoG) with the diffusion filtering for obtain a coherent vessel image. Multi-dimensional LoG gives initial details of all vessels, but there are minuscule vessels or vessels in complicated states including bending vessels, discontinuous vessels in the different regions require additional consistency through filtering by diffusion.
4. After vessel coherence, the final steps are segmented vessel images. For vessel image segmentation, we used double threshold as well as image reconstruction. The main purpose of using dual threshold based on histogram-based threshold selection is chosen to properly separate the vessels image from the background image, but some vessels are still missing due to noisy pixels. Image reconstruction is used to overcome this problem and obtained the well construction image.

The whole process is known as retinal vessel segmentation module based on multi-scale LoG filtering. There are few contributions in this research and these contributions are listed below:

1. We introduced the new steps to process retinal images by reducing noise because noise reduction almost improves vessel segmentation performance in later steps.
2. We have introduced new steps based on morphological steps compared to existing work and this step is used to handle the problem of the center of the light reflex and this small problem gives an indication of the variation of low contrast vessels and leads to implement the contrast enhancement method also.
3. We implemented a new image enhancement method based on homomorphic technique and Wiener filter to get a well-contrasted grayscale image.
4. Fourth major contribution of the proposed method is the implementation of a vessel coherent approach based on a Laplacian Gaussian (LoG) filter with diffusion filtering. This is one of the most innovative stages as it handles large, complicated, and small retinal vessels.
5. We have introduced a novel step for segmenting well vessel images with good quality. This technique relies on a dual thresholding and image reconstruction approach. Our developed technique has been observed to yield significantly superior results when compared to previously reported techniques.

This research paper has been organized into six additional parts. Section 2 involves a study of the previous research conducted in the field of retinal vessels. Section 3 provides a detailed account of the implementation of the proposed method. Section 4 provides the explanation of the database used in this work. Section 5 provides information on measurement parameters. Section 6 contains the analysis of the experimental results. Finally, Section 7 contains the conclusion and future research.

## 2 Related work

Autonomous segmentation of retinal vessels is crucial for analyzing retinal images using image processing techniques. In recent years, different researchers have utilized various techniques for the diagnosis and segmentation of retinal vessels based on fundus images. But there are still challenges for researchers to overcome. Retinal blood vessel segmentation is divided into two classes: Supervised method and Unsupervised method. Supervised category is further classified into SVM [28], CNN [29], ANN [22], GMM [17], while unsupervised category is further classified into morphological operation, the method based on filtering. In this section, we analysis existing supervised and unsupervised methods.

Xia et al. [30] implemented a supervised method and proposed a U-net based CTF-net for the precise segmentation of retinal vessels. The proposed method was evaluated in two different ways, with reference to organized retinal survey and computerized retinal images for vascular expulsion by the author. Typically, the disk and display each have accurate values of 94.34% and 94.97%. Thangaraj et al. [31] proposed an SVM-based method based on several steps: first convert the input image to an RGB channel. Then, a group of fundamental features such as local binary feature, gray level co-occurrence matrix feature, Hessian feature and Gobar filter is used to extract and compare the performance results, and RNN classifier is used to classify vessels and non-ship pixels. Finally, post-processing techniques were used to obtain a well-segmented retinal image.

Jin et al. [32] developed an automatic CNN-based model for segmenting retinal vessels. This technique used different resolutions of fundus images. Initially, they set up a systematic pipeline based on CNN. second stage, small resolutions images were used, their network is based on a collection of layers with addition of layers in common deep networks. Different models have used and compared their performance, including Deformable-ConvNet, M-VGG, M-ZF-Net, M-AlexNet, and LeNet. The segmentation of retinal vessels using Deformable-ConvNet was reportedly performed for the first time.This method was tested on the DRIVE and STARE databases and obtained an accuracy of 96.28% and 96.90% respectively.

Marín et al. [33] proposed a neural network model for pixel classification of fundus images. They used a multi-layered lookahead NN for training and classification and generated a 7-D vector for pixel representation composed of features based on momentum and grayscale invariants. They worked on the DRIVE and STARE databases and obtained satisfactory accuracy and sensitivity on these databases. However, the computation time was high, so it needed to be improved. A solution has been proposed by Cheng et al. [34] merging various context-aware features with a discriminative learning approach. They combined rich information using a random forest and utilized a cutting-edge method to achieve comparable results. They tested their method using STARE and DRIVE datasets and achieved mean classification accuracy values of 96.33% and 94.74%, respectively.

Azzopardi et al. [35] presented a technique for automatic segmentation of the structure of a vessel. This method uses a filter that selectively adjusts to detect B-COSFIRE-based ship trees. This method was validated on three datasets which indicated its effectiveness in minimizing computational complexity and achieved sensitivity of 76.5%, 77.1% and 75.8% on DRIVE, STARE and CHASE_DB1 respectively. Lazar et al. [36] proposed a segmentation technique relies on merging a region-expanding process with a hysteresis thresholding method that inturn relies on the feedback of nearby pixel similarity vectors. Additionally, Soomro et al. [37], presented an enhancement technique to segment a retinal blood vessel from abnormal fundus images. They used PCA to convert the RGB image into a single grayscale image to greatly improve the sensitivity of the ships array. Anisotropic scattering used for vessel array normalization and their method achieved an accuracy of 0.95.

The method of Khawaja et al. [38] has been shown to be effective in improving the frame segmentation performance of retinal vessels using state-of-the-art speckle denoising, another type such as based deionising. Charles et al. [39] used PPB denoising technique, it was mainly used to improve detection of large vessels, but this scheme also detected small and large vessels independently but their method drops many tiny vessels.

Da Rocha and Douglas Abreu [40], proposed a technique to improve the contrast of retinal fundus images based on an image processing algorithm. This method improved their performance and level of accuracy through two modules, namely: pre-processing and segmentation steps. This technique, which relies on fractal dimensions and morphological correction, has been suggested [41] for enhancing the background contrast of vessels of retina in fundus images. Initially, pre-processing was used to separate the green channel from the RGB image and the light reflection is eliminated by the morphological factor in conjunction with the linear structural components. Next, multi-threshold and fractal dimensions are used to separate the blood vessels from the background. This technique was validated on the STARE, DRIVE, and HRF database.

Neto et al. [42] proposed a method based on a local threshold on the cumulative distribution to improve the contrast of the raw image and then converted it to a green channel. The results are then enhanced with morphological techniques that minimize image noise. This method was tested on the DRIVE and STARE databases and obtained an accuracy of 78.1% and 87%, and a sensitivity of 78.4% and 86.6% respectively. Moreover, Wang et al. [43] reported an image enhancement method to improve the low contrast of retinal blood vessels. This method simultaneously overcomes various fundus image problems such as uneven illumination, detail enhancement, noise, and blurring. This methodology considers raw images in three retinal image RGB channels to compensate for non-uniform background based on weighted blending tactics to reduce noise and artifacts.

After studying the different methods, all of these methods have the disadvantage of not properly tracing tiny vessels and having lower sensitivity, meaning that small vessels are not detected accurately. Another main drawback is the difficulty in achieving improvement. In the proposed methods, we implement a method that overcame these problems and a detailed explanation of this method is given in the next section.

## 3 Proposed method

**Fig 1:**
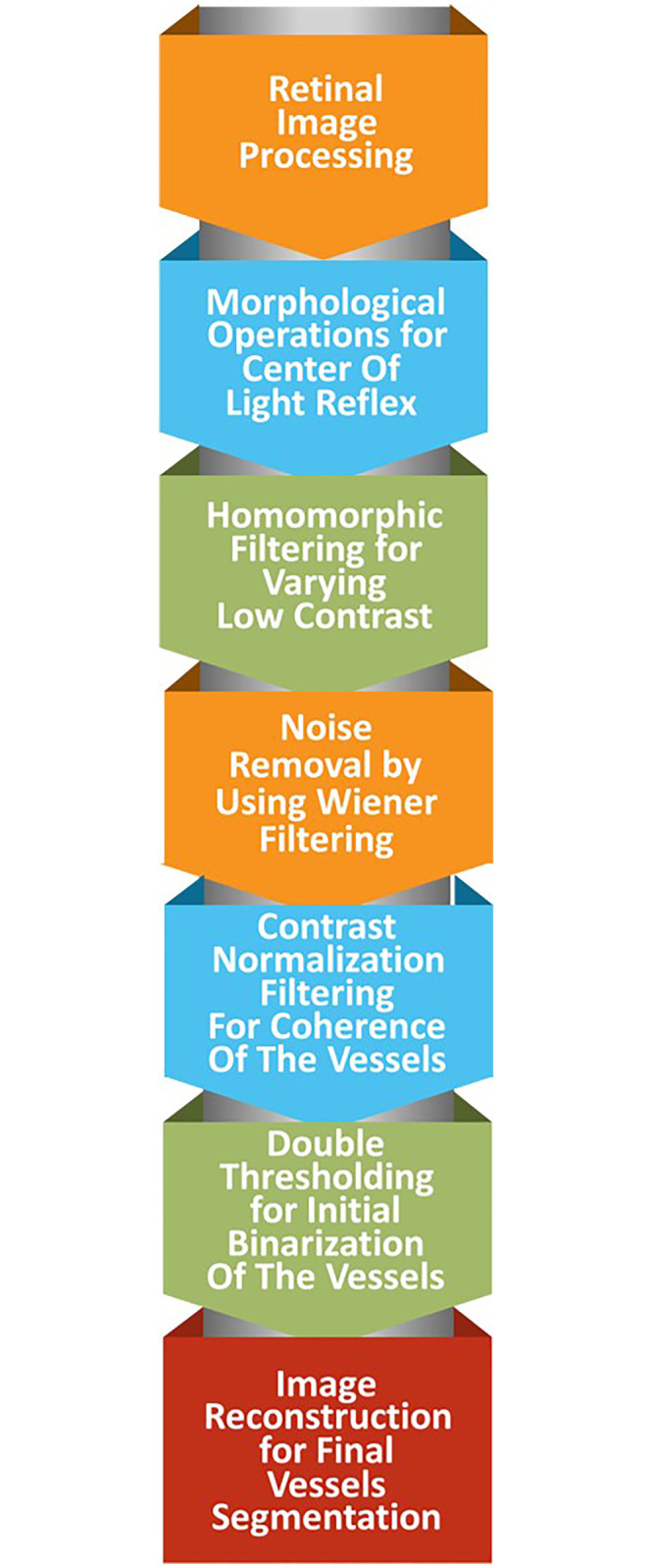
The proposed model.

The proposed methods contain the following new steps as shown in the Fig 1. A detailed explanation of each step is provided below.

### 3.1 Retinal image processing

**Fig 2:**
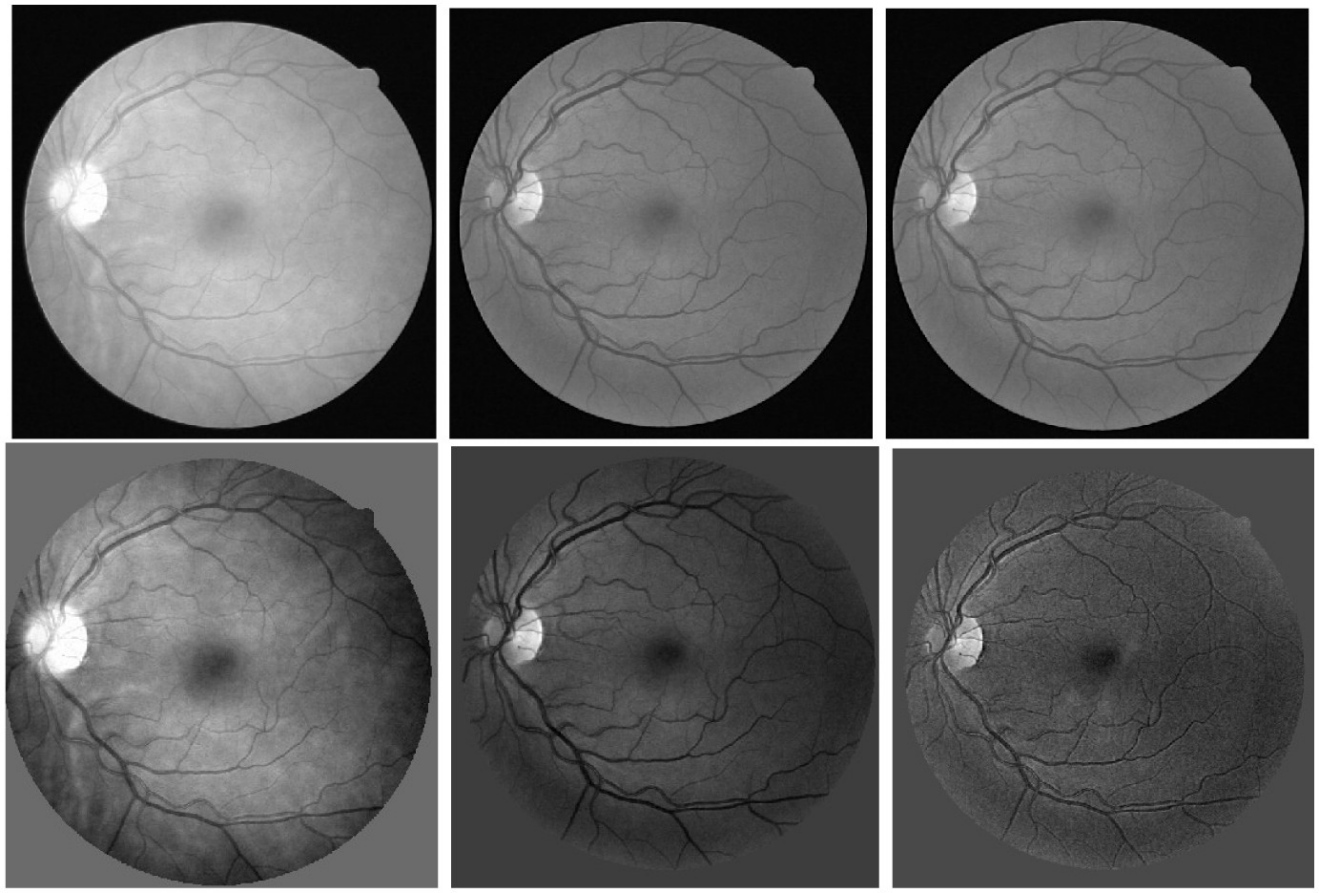
The first line represents the RGB images and the second row represents their images converted to grayscale.

The first step of the proposed method is the process of retinal images, and it involves the problem of uneven illumination and noise caused by the center of the light reflex. To handle all mentioned issues, we need to implement the new image enhancement technique, and the new image enhancement techniques have a direct impact on the vessel segmentation process. The first step is crucial for implementing the enhancement technique that’s the input image process. The retinal images are the red-green-blue (RGB) image. Color images take time to process, and we convert the retinal image to RGB channel to observe each channel. We convert RGB images to grayscale which gives a clear analysis of the center of the light reflex as shown in the Fig 2 and the grayscale conversation also reduces the time computation. This helps us in the next steps to deal with uneven lighting as well as noise.

### 3.2 Morphological operation for removal centre light reflex

**Fig 3:**
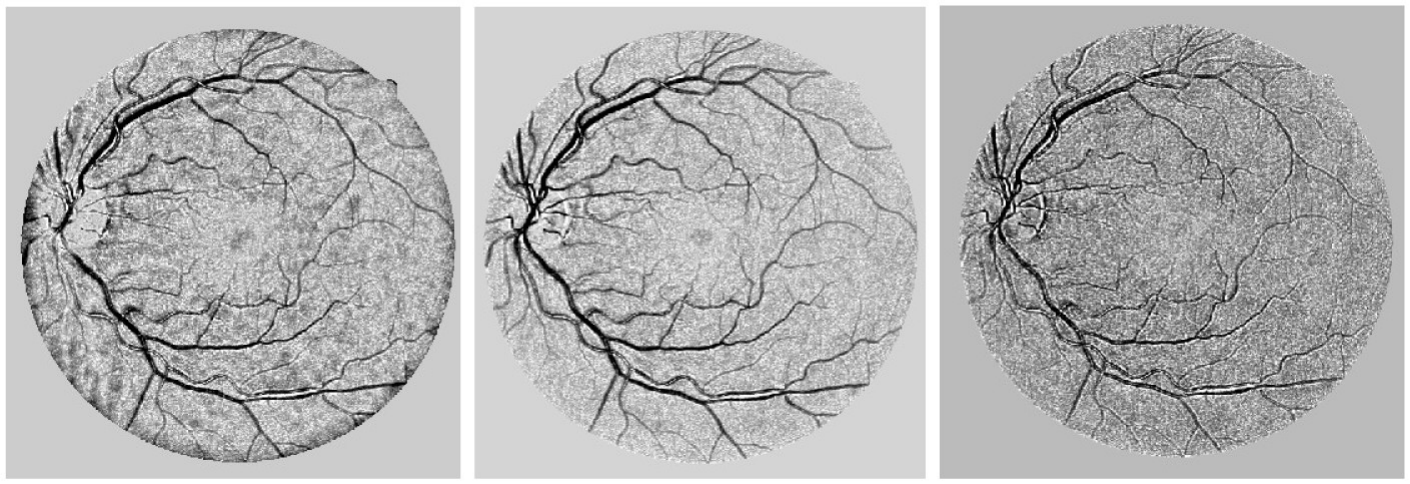
Output of morphological tactics on each channels(Red, Green and Blue).

Morphological tactics are used to deal with the central light reflex problem and make background and foreground pixels uniform. We used the bottom hat operation on each channel initially to analyze the center of the light reflex effect on the vessels and we observe that there is a suppression of the lighting which gives the contrast variation but there is some impact of lighting on the background which makes it difficult to analyze foreground pixels. Then after applying the top hat operation and subtracting its output from the output of the top hat operation, this clearly gives an analysis of the foreground pixels (vessels pixels) versus their foreground pixel and background with light reflex suppression. The mathematical representation of these operations in the set of equations from Eqs 1 to 3 below and the output image is shown in the Fig 3.

$$\begin{array}{c} T_{b}\left(f\right)=f•b-f. \end{array}$$ (1)

The closing operation is denoted by a *bullet*.

$$\begin{array}{c} T_{t}\left(f\right)=f-f^{\circ }b. \end{array}$$ (2)

The opening operation is denoted by *circ*.

$$\begin{array}{c} T_{output}\left(f\right)=T_{b}\left(f\right)-T_{t}\left(f\right). \end{array}$$ (3)

### 3.3 Homomorphic filtering for varying-low contrast

Retinal images are severely affected by varying-low contrast; it is very important to have a uniform contrast of background and foreground to observe the details of the image. The homomorphic filter [44, 45] is used for retinal fundus images to suppress uneven illumination. Homomorphic filtering is a frequency domain approach and mainly contains the imaging model. As image formation depends on two parameters, illuminance and reflectance, image formation is the product of illuminance and reflectance. The illuminance has a slow spatial variation because it is a low frequency component, the reflectance has sudden variations in the spatial domain and is represented as a high frequency component.

$$\begin{array}{c} F\{I(x,y)\}=F\{L(x,y)\}.*F\{R(x,y)\}. \end{array}$$ (4)

Consider the Eq 4 which represents the image formation model in the Fourier transform, the image (*I*) is the product of two components, illumination (*L*) and reflectance (*R*) of the image. The main task is to estimate the reflectance image; a logarithm of the image has been applied to facilitate the calculation, as shown in the Eq 5.

$$\begin{array}{c} F\{\text{ln}(I(x,y))\}=F\{\text{ln}(L(x,y))\}+F\{\text{ln}(R(x,y))\}. \end{array}$$ (5)

The image can be exploited on the different frequency domains with the introduction of the *H* filter

$$\begin{array}{c} M\left(u,v\right)=F_{L}\left(u,v\right)H\left(u,v\right)+F_{R}\left(u,v\right)H\left(u,v\right), \end{array}$$ (6)

where *M*, *F*_*L*_ and *F*_*R*_ are the Fourier transforms of ln(F), ln(L) and ln(R), and (*u*, *v*) are the coordinates in the frequency domain. If the high pass filter is then applied, the reflectance component would be obtained with the elimination of the illumination component. The inverse Fourier transform with an exponential transform is used to obtain the final reflectance image.

$$\begin{array}{c} k(x,y)=e^{m(x,y)}=e^{F{}^{-1}\left\{M(u,v)\right\}}. \end{array}$$ (7)

The output of the enhanced filter (Gaussian high-pass filter) used for homomorphic filtering is represented mathematically by

$$\begin{array}{c} \begin{array}{c} H\left(u,v\right)=\left(\gamma_{H}-\gamma_{l}\right)\left[1-e^{-(D^{2}\left(u,v\right)/{D_{0}}^{2}}\right]+\gamma_{l} \end{array} \end{array}$$ (8)

where *γ*_*H*_ and *γ*_*l*_ compute respectively the maximum and minimum values of the filter. *D*_0_ is the center of the image in the frequency domain, *D*(*u*, *v*) is the distance between coordinates (*u*, *v*) and *D*_0_.

**Fig 4:**
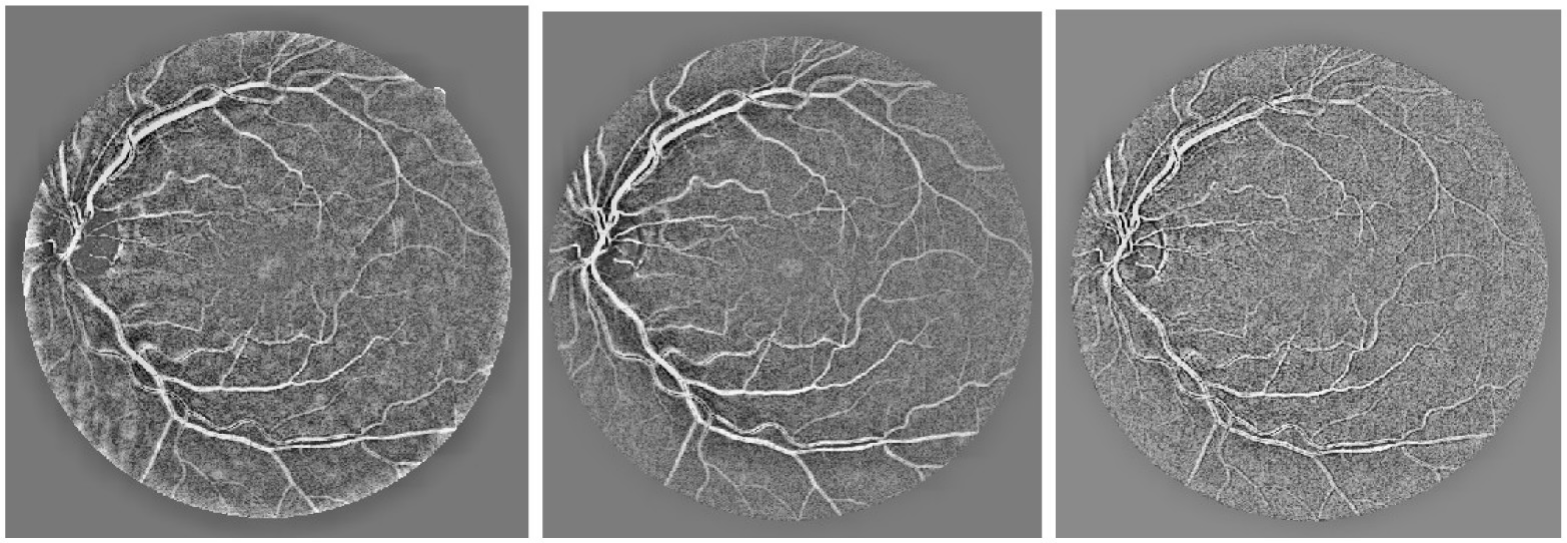
Homomorphic filter output on each channel.

The main task of using the homomorphic filter is to achieve uniform contrast between the retinal blood vessels against the background. The filtered homomorphic output of each gray RGB channel is shown in Fig 4. The impact of the non-uniform background suppression part of the proposed method is to overcome two main limitations (noise and uneven illumination) to achieve accurate vessel detection. But still, the tiny blood vessels are not seen clearly due to the noise and we have used Wiener filtering in the following sections to remove the noise.

### 3.4 Noise removal by using Wiener filtering

Wiener filtering is the type of filtering applied to the nosiy image based on a statistical measurement of the statistical parameters. The two statistical measures on which a wiener filter depends in a region with a defined window of the image *M* × *N* are the variance and the mean. The Wiener filtering operation is applied to the retinal image after homomorphic filter for noise identification and the operation of Wiener filtering is defined below.

**Fig 5:**
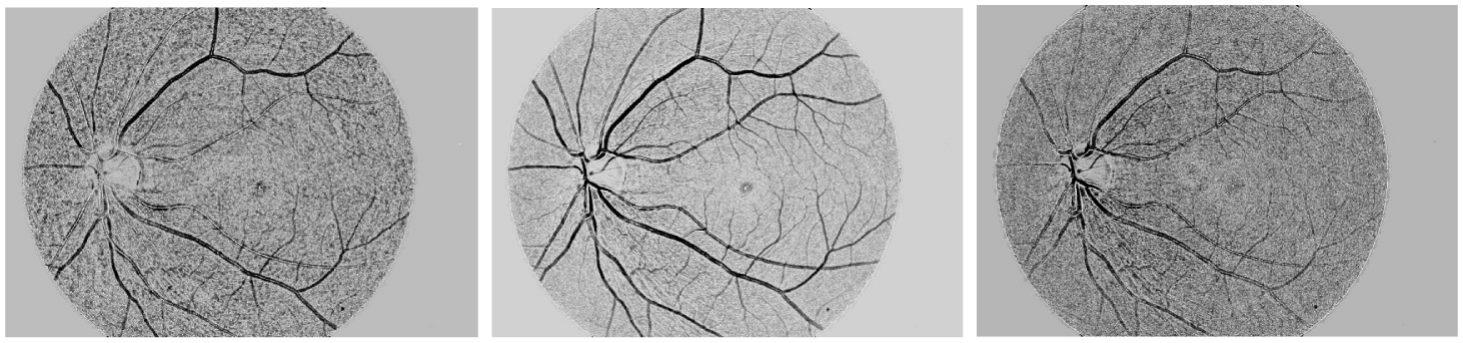
Output of Wiener filter on each RGB channel.

The Wiener filter belongs to a class of filters called adaptive filters, which modify the characteristics of each pixel in the image based on their values. These types of filters can remove Gaussian noise. The minimum mean squared error filter, also known as the Wiener filter, belongs to the class of such filters. It is customized based on the image local variance. When there exists a significant variance, the Wiener filtering operation results in negligible smoothing. When there exists a significant variance, the Wiener filter carries out a greater smoothing operation. A filter that has a linear response of the same type has less sensitivity compared to an adaptive filter. The adaptive filter will preserve the image corners and various high frequency areas. Adaptive Wiener uses a pixel-by-pixel to get well denoised image. This method uses the statistics of each pixel from the local neighborhood. The adaptive Wiener filtering operation works in three stages:

1. The image contained in the noise is averaged accordingly to provide the mask. The Eq 9 is a representation of the math problem for this step
  $$\begin{array}{c} \mu =\frac{1}{\text{NM}}\sum_{n_{1}=1}^{N}\sum_{n_{2}=1}^{M}I(n_{1},n_{2}). \end{array}$$ (9)
  For this calculation, the image’s local neighborhood *NbyM* is employed for each pixel.
2. The mask is obtained by computing the variance of the noisy image. The Eq 11 is a representation of the mathematical problem for this step.
  $$\begin{array}{c} \mu =\frac{1}{\text{NM}}\sum_{n_{1}=1}^{N}\sum_{n_{2}=1}^{M}I^{2}(n_{1},n_{2})-\mu^{2}. \end{array}$$ (10)
  For this calculation, the image’s local neighborhood *NbyM* is employed for each pixel.
3. The adaptive Wiener creates a pixel-by-pixel Wiener filter using these estimates. Eq 3 is a representation of the math problem in this step.
  $$\begin{array}{c} F(n_{1}n_{2})=\mu +\frac{\sigma^{2}+v}{\sigma^{2}}(I(n_{1},n_{2})-\mu ). \end{array}$$ (11)
  Accordingly, The variance of the noise is denoted by v. On the other hand, In the absence of a given noise variance, the adaptive Wiener filtering operation will utilize the mean of all evaluated local variances. The resulting images of gray RGB adaptive Wiener filtering are shown in Fig 5.

### 3.5 Contrast normalization filtering for coherence of the vessels

Vessels are commonly detected by approximating them with a geometric shape called ridges, which are thin lines that are darker or brighter than their surroundings. To detect ridges and suppress all other structures in an image, the major eigenvalue is calculated at each pixel. The major eigenvalue is a second-order derivative oriented in a specific direction and needs to be pre-smoothed with an anisotropic Gaussian function to enhance noise immunity. This process results in an elongated second-order Gaussian detector known as the 2nd order multi-dimensional Laplacian Gaussian Detector.

The detector works by utilizing three parameters: length (*σ*_*u*_), width (*σ*_*v*_), and orientation. To maintain elongation, the length parameter *σ*_*u*_ is required to be a multiple of width *σ*_*v*_, with the factors having values from the set 0.5, 1, 1.5, 2, 2.5, 3, 3.5. The width parameter *σ*_*v*_ is chosen from the set 4, 5. The maximum response is selected from all possible sets of values for length, width, and orientation. To identify the maximum response, a generalized two-dimensional Gaussian function is applied, encompassing all possible combinations of length, width, and orientation values. The function is defined in Eq 12.

$$\begin{array}{c} g(u,v)=\frac{1}{2\pi \sigma_{u}\sigma_{v}}{\text{exp}}^{\left(-(\frac{u^{2}}{2\sigma_{u}^{2}}+\frac{v^{2}}{2\sigma_{v}^{2}})\right)}. \end{array}$$ (12)

From this set, the maximum response is selected. By utilizing two independent parameters, *σ*_*u*_ and *σ*_*v*_, the generalized Gaussian function can be expressed. Taking the second-derivative of this function with respect to only *u* yields the following equation:

$$\begin{array}{c} g_{uu}(u,v)=\frac{1}{2\pi \sigma_{u}^{5}\sigma_{v}}(u^{2}-\sigma_{u}^{2}){\text{exp}}^{-(\frac{u^{2}}{2\sigma_{u}^{2}}+\frac{v^{2}}{2\sigma_{v}^{2}})}. \end{array}$$ (13)

**Fig 6:**
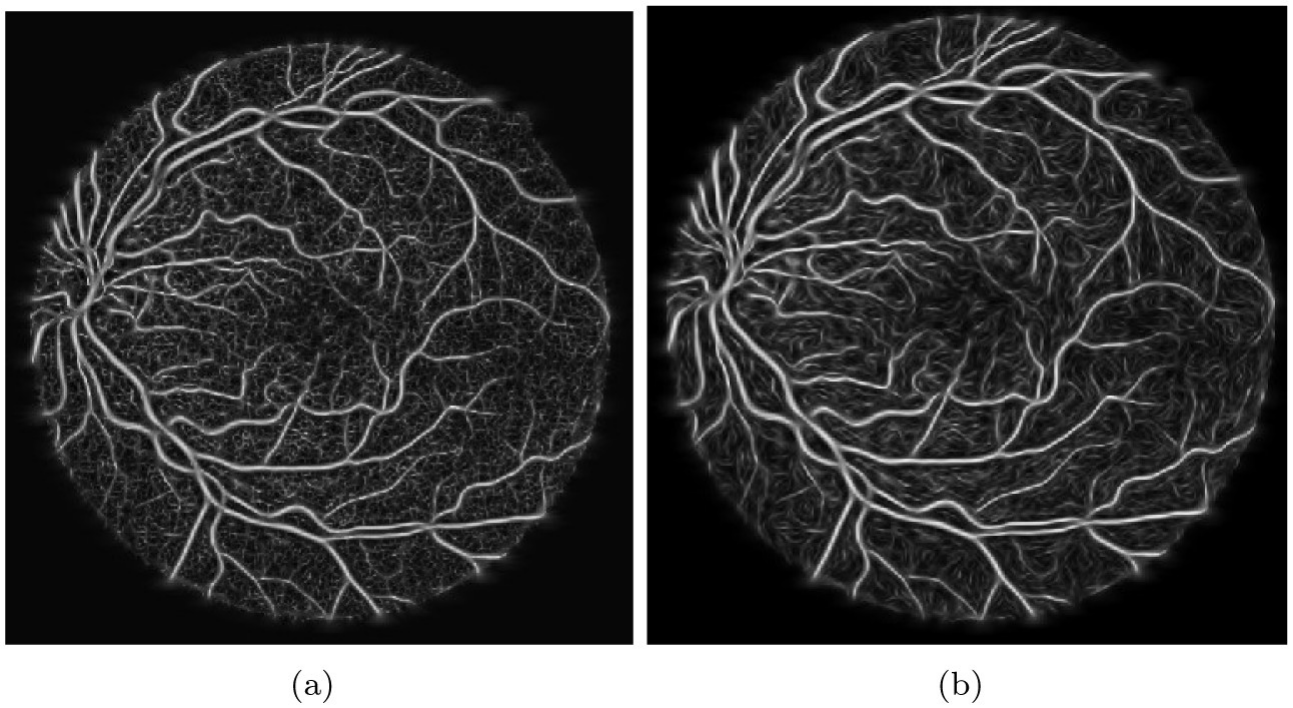
Output of the 2nd order multi-dimensional Gaussian derivative identifier, where (a) denotes the primary output with the maximum value, and (b) represents the normalized factor applied to the initial output.

To rotate the discrete kernel in a specific orientation, the equations *u* = *x* cos *θ* − *y* sin *θ* and *v* = *x* sin *θ* + *y* cos *θ* are utilized. The response of the oriented kernel is then normalized by multiplying it with a factor of $\sigma_{u}^{\alpha }\sigma_{v}^{\beta }$, as illustrated in Fig 6(a).

In the study by Lindeberg [46], optimal values for *α* and *β* were determined for ideal ridge patterns, with values of *α* = 1.5 and *β* = 0.5. However, since the focus of our study is on enhancing the detector intensity for small-width vessels with lower contrast, a more suitable choice for our database images would be *α* = 1 and *β* = 0.5. By using these scale-normalization factors, the maximum response for each pixel is determined by examining all possible combinations of length, width, and orientation.

After comparing the contrast across the blue, green, and red channels, it was found that the green channel provides the best visualization of the vessels due to its superior contrast. Therefore, the green channel was chosen for further analysis. Although the suggested measures improved the overall contrast of the retinal image, there were still discontinuities in the vessel outflows that required attention. To address this issue, a 2nd order multi-dimensional Laplacian Gaussian detector was employed to bridge the voids in the connecting vessels and generate coherence in the vessel regions, as depicted in Fig 6.

Despite filling the gaps, the larger vessels still exhibit good contrast against the tiny capillaries since the tiny vessels do not possess the same level of coherence as the background. However, binarizing the vessels in their current form remains challenging. To address this issue, method [47] was utilized, which involves applying an anisotropically oriented diffusion filter operation to enhance the consistency of small vessels.

**Fig 7:**
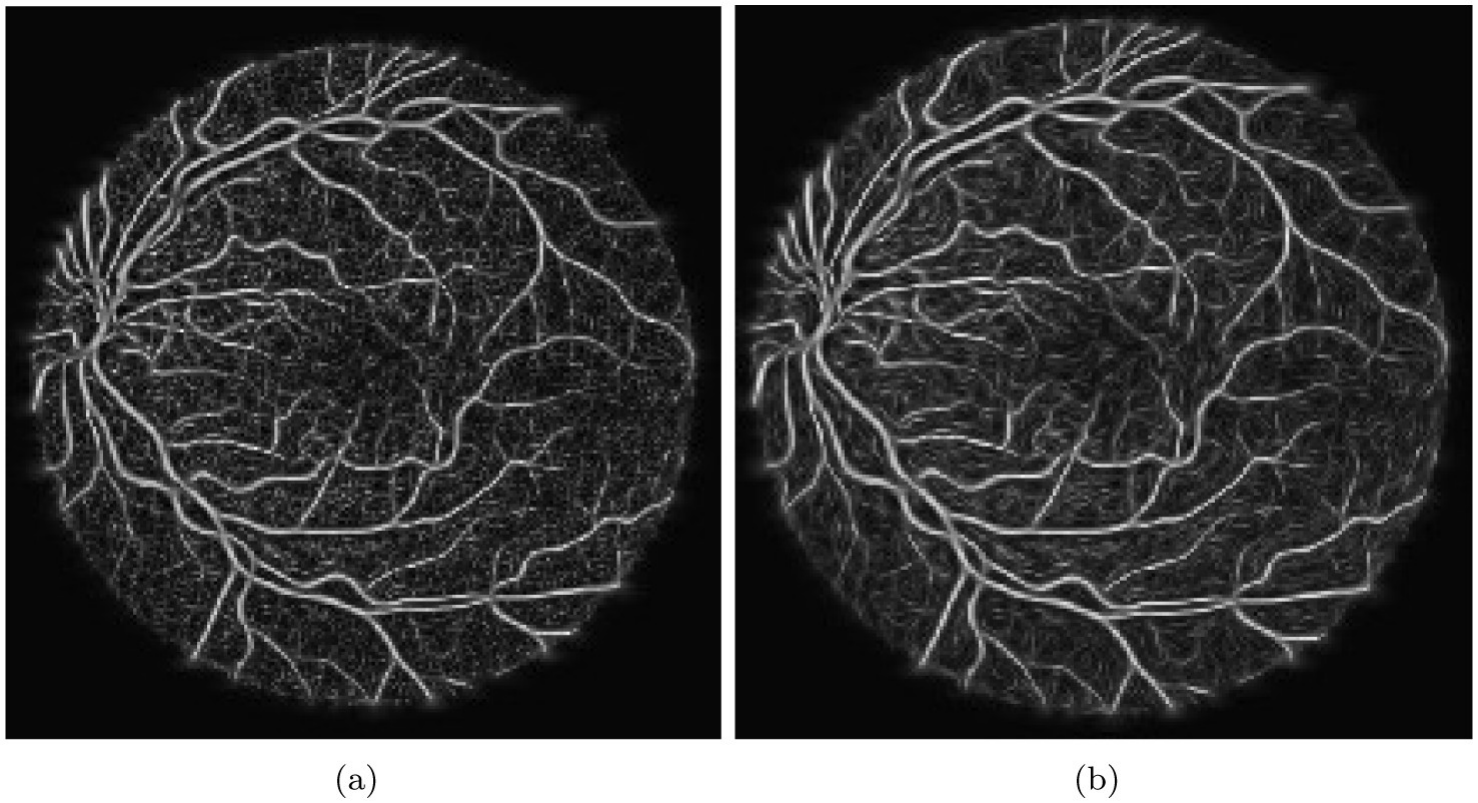
Oriented Diffusion Output, where (a) represents the retinal image prior to applying the Coherent Anisotropic Diffusion filtering operation, and (b) represents the proposed scheme of Coherence Anisotropic Diffusion filter.

The process of anisotropic diffusion filtering involves an iterative approach that begins with the primary retinal images and gradually smooths out the structures with each step. The objective is to obtain the optimal filtered image through a stopping criterion, which determines the end point of the diffusion filtering process. In this regard, a stopping criterion proposed in [48] has been utilized. It is based on monitoring the rate of change in the spatial entropy value of the retinal vessel image over the iterations. When the entropy value reaches a stable state, the diffusion filtering process is stopped. Fig 7 presents the outcome of an anisotropic diffusion filtered image using the aforementioned stopping criterion. The filtered image exhibits a reduced level of noise and better-defined vessel edges, making it easier to analyze and extract useful information.

### 3.6 Double thresholding for initial binarization of the vessels

**Fig 8:**
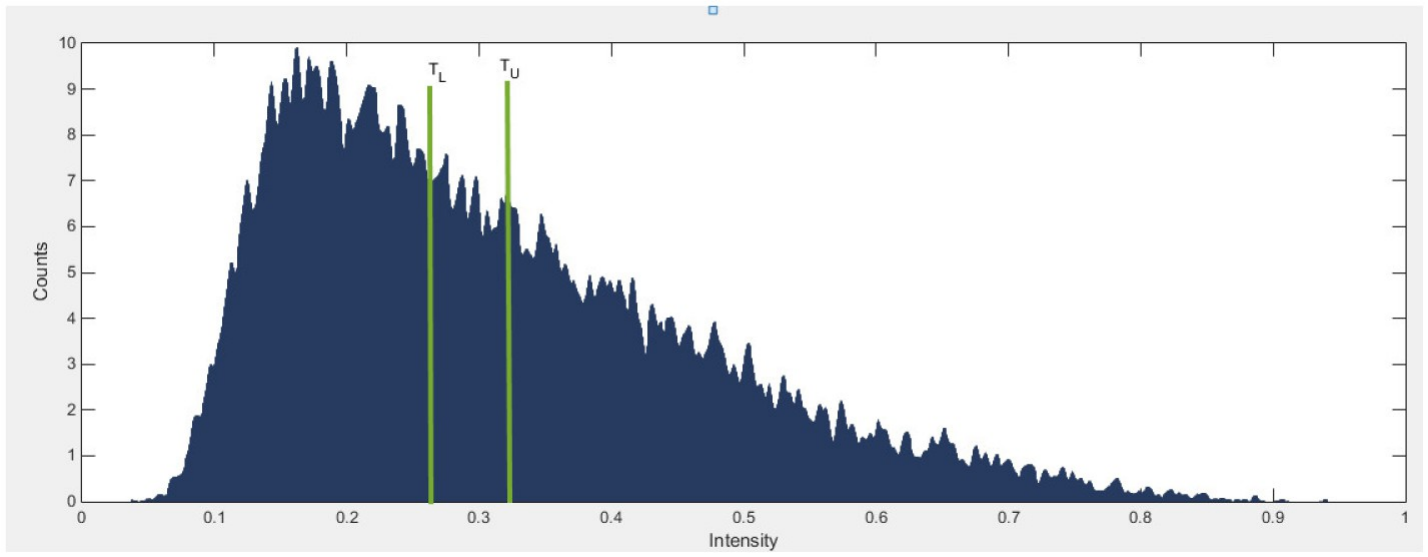
The histogram identified two vertical bars to serve as the two thresholds. *T*_*U*_ is obtained by subtracting 0.7 times the standard deviation from the mean value of the image histogram, while *T*_*L*_ is obtained using the mean value of the edge-based histogram.

**Fig 9:**
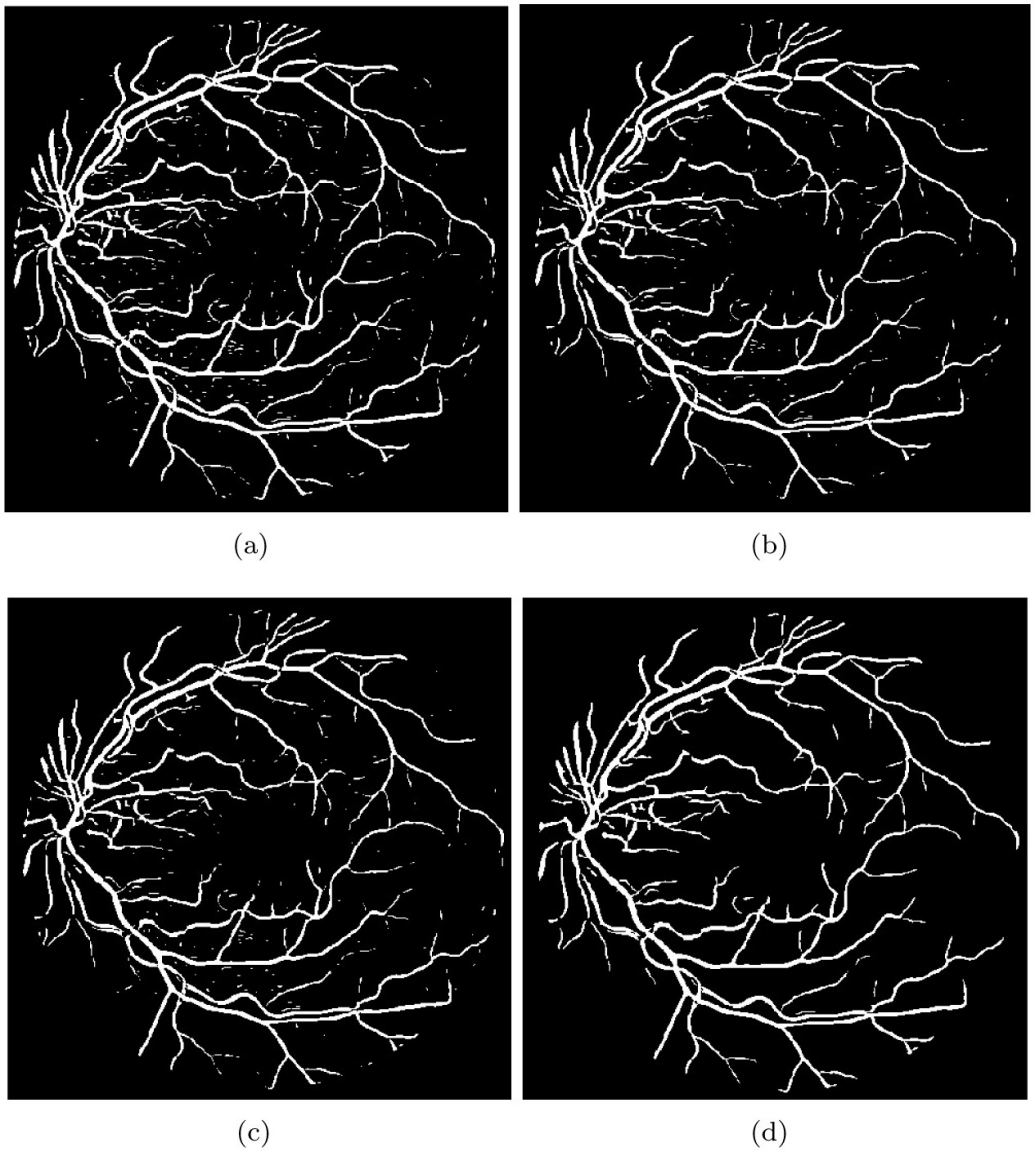
Displays the various images obtained during the segmentation process. The mask image and marker image are presented in Fig(a) and (b), respectively. The morphologically reconstructed image is depicted in Fig (c), while the finilized binary image of segmented vessels is illustrated in Fig (d).

Upon achieving coherence of the retinal vessels, the final step involves segmenting the retinal vessels. We utilized the double threshold imaging method to generate a segmented image of the vessels. We used double threshold techniques based on the selection of histograms of values. Threshold values are selected using morphological reconstruction techniques, and the main purpose of morphological reconstruction techniques is to create the binary image. The binarized version of the images was created by combining the mask and marker, which were acquired from the histogram of image coherence, as depicted in Fig 8. As, depicted in Fig 9(a), to generate the mask image, the mean value of the image was computed by analyzing its histogram. Fig 9(b) also depicts the marker image that was acquired by subtracting 0.7 times the standard deviation from the mean value of the image.

### 3.7 Image reconstruction for final vessels segmentation

Then we used the mathematical operation known as morphological reconstruction to acquire segmented vessels (The image reconstructed from the process is displayed in Fig 9(c)). However, the segmentation of tiny vessels is often not accurate due to the presence of noisy areas. To address this, we utilized a basic image processing technique that eliminates small areas with less than 70 pixels. The resulting binary or is presented in Fig 9(d).

## 4 Database and measuring parameters

Two publicly available databases are used by many researchers to validate the performance of proposed methods, including the impact of Wiener filtering and binary techniques. This section contains the information of the databases and parameters used to validate the performance of our developed technique.

### 4.1 Databases

We used the two accessible datasets, Structured Analysis of the Retina (STARE), and Digital Retinal Images for Vessel Extraction (DRIVE) which are often used by researchers to validate their proposed methods. The databases confirm the effectiveness of the technique we propose. The DRIVE dataset has 40 images. The images were obtained using a Canon CR5 non-mydriatic 3-CCD camera made by Canon, Tokyo, Japan, as part of an ocular health screening campaign in the Netherlands. The field of view (FOV) of the camera was 45 degrees. The captured images comprise two groups, namely testing and training, with a resolution of 768 × 584 pixels.. Each group contained 20 images with their mask and ground truth image.

The STARE database contains 20 images, and 75% of these images contain disorders, providing a favorable opportunity to validate the capabilities of our proposed method. These images are captured using A TopCon TRV-50 Fundus Camera A (Topcon, Tokyo, Japan) and it contains the resolution of 605 × 700 pixels of each image.

## 5 Measuring parameters

We have used the most common measurement parameters because this gives possibilities of comparison with existing work. The parameters used for evaluating the performance of the proposed method are area under the curve (AUC) specificity (Spec), accuracy (AC), and sensitivity (Sen). Sensitivity and specificity tell us about the imbalance of vessel and non-vessel classification pixels and give us a good idea of small vessel fall off and improvement in sensitivity shows improvement in small vessel segmentation. AC gives overall information on vessel segmentation. But there is still a need for a parameter that provides information about the misclassification of pixels belonging to vessels as non-vessel and vice versa during segmentation. The AUC parameter provides information on the classification of true non-vessel pixels (specificity) and true vessel pixels (sensitivity). The AUC is calculated using the formula *AUC* = (*Se* + *Sp*)/2, but a more appropriate parameter for evaluating performance would be the accurate the true classifications of the vessels.

## 6 Experiment set up and results analysis

This section contains the evaluation of the analysis of the experimental results for our proposed segmentation model. The experimental configuration is based on MATLAB 2018 on core i7 at 3.6 GHz with 16 GB of RAM.

### 6.1 Performance analysis of proposed method

This section contains the evaluation of the analysis of the experimental results for our proposed segmentation model. The experimental configuration is based on MATLAB 2018 on core i7 at 3.6 GHz with 16 GB of RAM.

### 6.2 Performance analysis of proposed method

The performance of the segmentation method was thoroughly evaluated, and the results are presented in Table 1. The impact of improvement techniques on the performance was analyzed, particularly through the utilization of Homomorphic filtering and Wiener filtering as enhancement filters. These filters significantly influenced the overall performance of the developed technique. By examining Table 1, it becomes apparent that the improvement-based segmentation model exhibited significantly better performance compared to the segmentation model without any improvement techniques.

**Table 1:** Analysis of segmentation method’s performance.

Dataset	STARE		DRIVE	
Attribute	Without Enhancement	With Enhancement	Without Enhancement	With Enhancement
AUC	0.623	0.885	0.716	0.895
AC	0.481	0.951	0.512	0.959
Sp	0.682	0.968	0.781	0.977
Se	0.571	0.801	0.652	0.813

**Fig 10:**
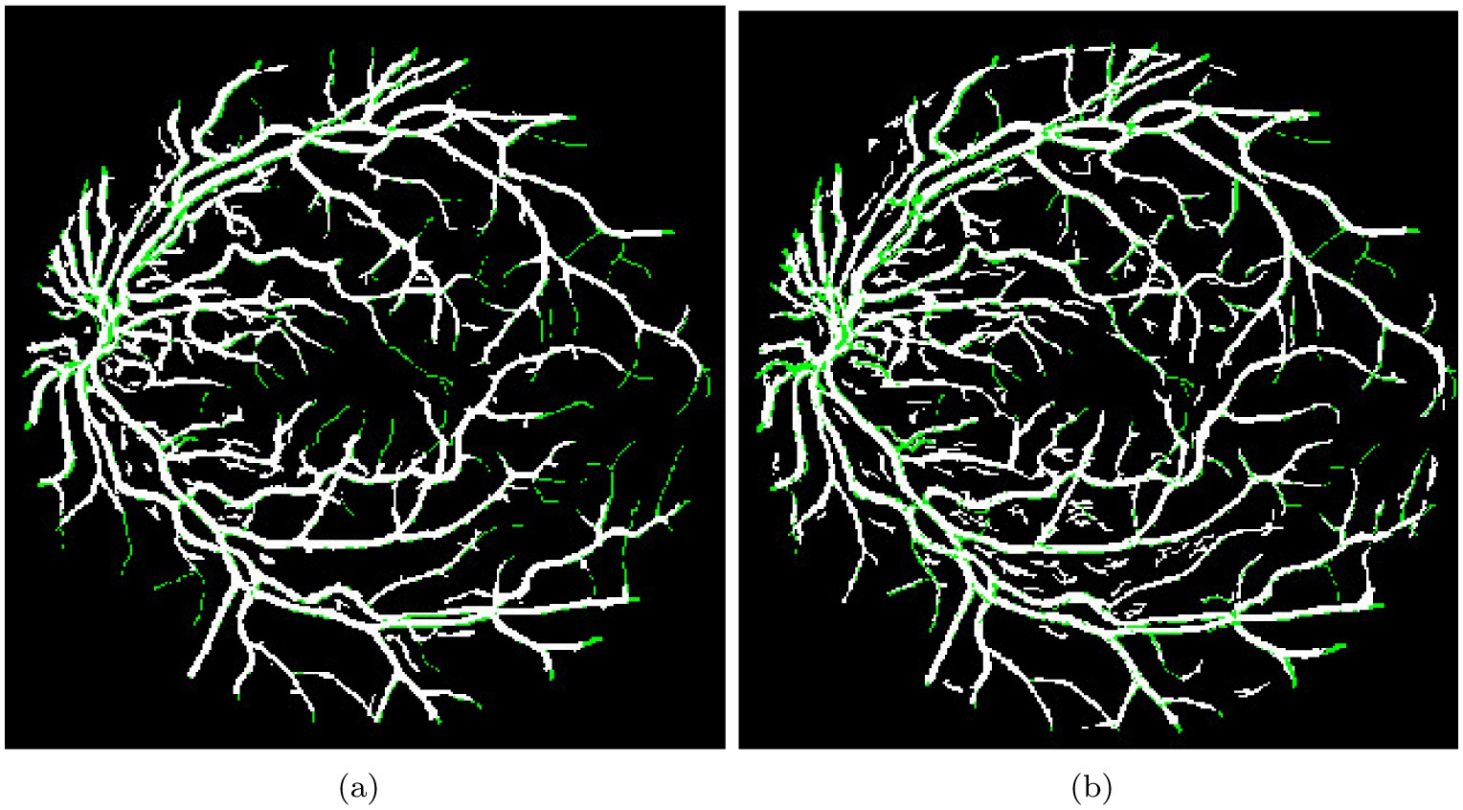
(a) Segment Image without Enhancement (Green lines indicates the missing Vessels (b) Segment Image with Enhancement. Note: Green lines indicates the missing Vessels.

The results indicate that the segmentation image obtained using the improvement-based model accurately detects small vessels, almost on par with the ground truth image. This observation is evident when comparing the vessels in the output images with and without segmentation, as shown in Fig 10.

The comparison between the output images with and without segmentation clearly demonstrates the effectiveness of the proposed segmentation method. The presence of the segmentation step greatly enhances the visibility and differentiation of the vessels, allowing for a more accurate identification and analysis. It is particularly noticeable in the detection of small vessels, where the segmentation model excels in capturing their intricate details. There are several avenues for future work and improvements. One crucial aspect to address is the detection and segmentation of missing vessels. The current method primarily focuses on the segmentation of visible vessels, and the accuracy in capturing missing vessels needs to be further improved. Advanced techniques, such as incorporating deep learning models, could be explored to enhance the identification and segmentation of these missing vessels, thereby providing a more comprehensive and accurate vessel analysis. Furthermore, the performance of the proposed method should be validated on larger and more diverse databases containing a wide range of retinal images. This will help assess the generalizability of the method and ensure its effectiveness across different populations and clinical scenarios. In addition, future research could explore the integration of the proposed segmentation method into a practical software tool for automated eye disease analysis. This would involve refining the method’s implementation, optimizing its computational efficiency, and considering the integration within existing clinical workflows or software systems. Overall, while the presented results demonstrate the effectiveness of the improvement-based segmentation model, further enhancements are necessary, particularly in capturing missing vessels. Validating the method on larger datasets and developing a practical software tool would significantly contribute to advancing retinal vessel analysis and supporting ophthalmologists in diagnosing ocular abnormalities.

### 6.3 Evaluation of the method’s accuracy on challenging images

**Fig 11:**
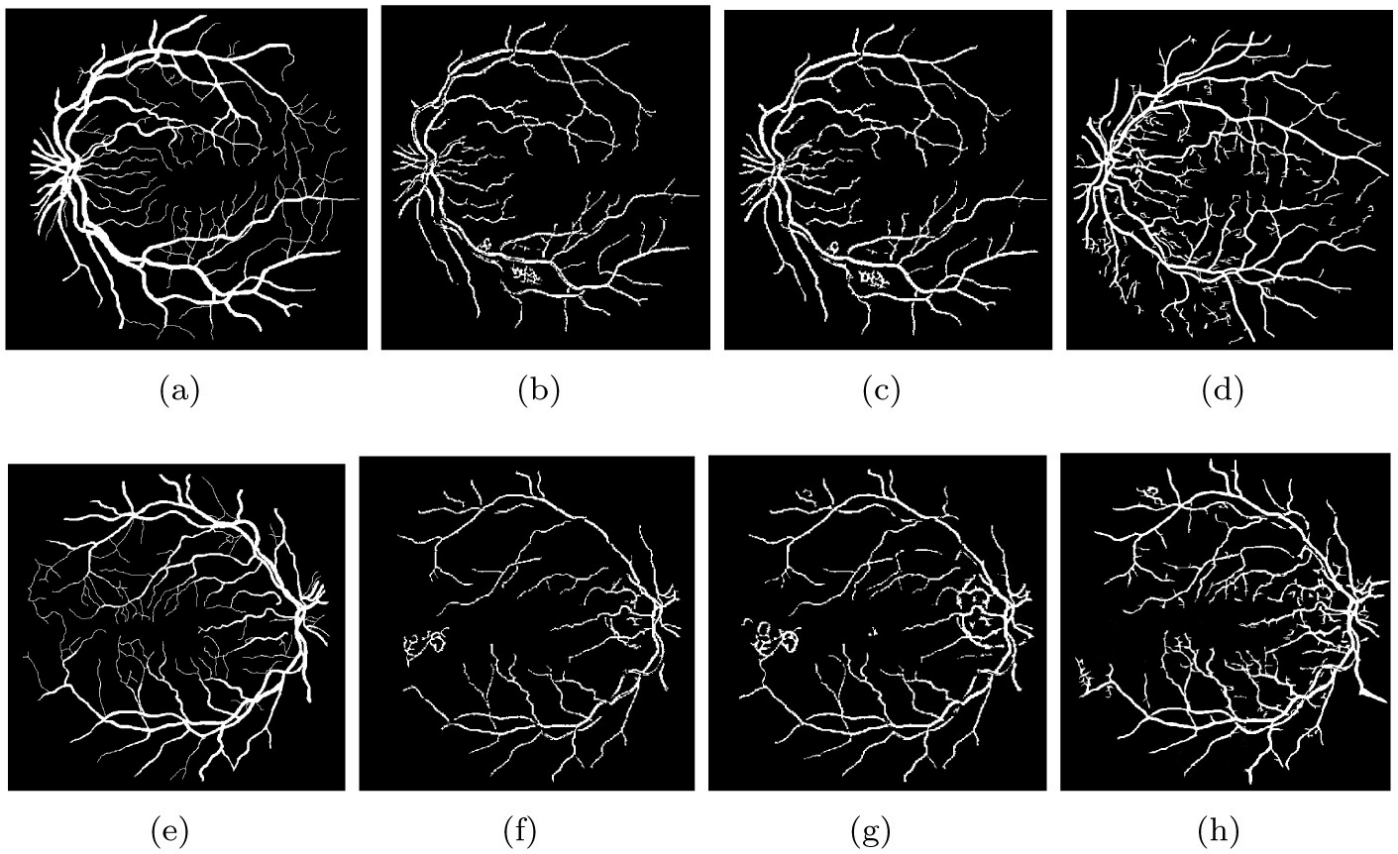
Our proposed method was evaluated on images with pathologies, and the results are presented in Fig (a and e), which depict the ground truth images. Fig (b and f) show the results obtained by the Nuygen method on these images, while Fig (c and g) show the results obtained by the Hou method. Fig (d and h) shows the results obtained by our proposed method on these images.

The STARE and DRIVE datasets are two well-known datasets in the field of medical image analysis, which contain images of the retina with roughly 50% pathological and other abnormalities. Detecting the vessels of the retina on these images is a challenging task, mainly due to the presence of abnormalities and noise. As a result, these images are often referred to as challenging images. To address this challenge, we proposed a new method for accurately segmenting the vessels of the retina on these challenging images. Our proposed method was tested on these images, and the results presented in Table 2 indicate that our method outperformed other methods by a significant margin, accurately segmenting the vessels of the retina. To better illustrate the effectiveness of our proposed method, we displayed the output images of the proposed algorithm on pathology-contained challenging images in Fig 11. The output images of our proposed method are shown in Fig 11(d) and 11(h), which demonstrate the successful segmentation of the vessels of the retina even in the presence of significant abnormalities and noise. Furthermore, to further evaluate the effectiveness of our proposed method, we compared the output of our method with two novel methods, namely the Nguyen method [49] and the Hou method [50]. The output images of Nguyen and Hou methods are shown in Figs 11(b), 11(c), 11(f) and 13(b). The comparison clearly shows that our proposed method outperformed both methods, particularly in detecting the tiny vessels. In this analysis, our proposed method demonstrates excellent performance in accurately segmenting the vessels of the retina on challenging images, where other methods fail. The proposed method can be potentially used for automated screening and diagnosis of retinal diseases.

**Table 2:** Challenging images performance assessment.

Database	*Se*	*Sp*	*Ac*	*AUC*
DRIVE	0.821	0.952	0.964	0.887
STARE	0.803	0.959	0.961	0.881

**Fig 12:**
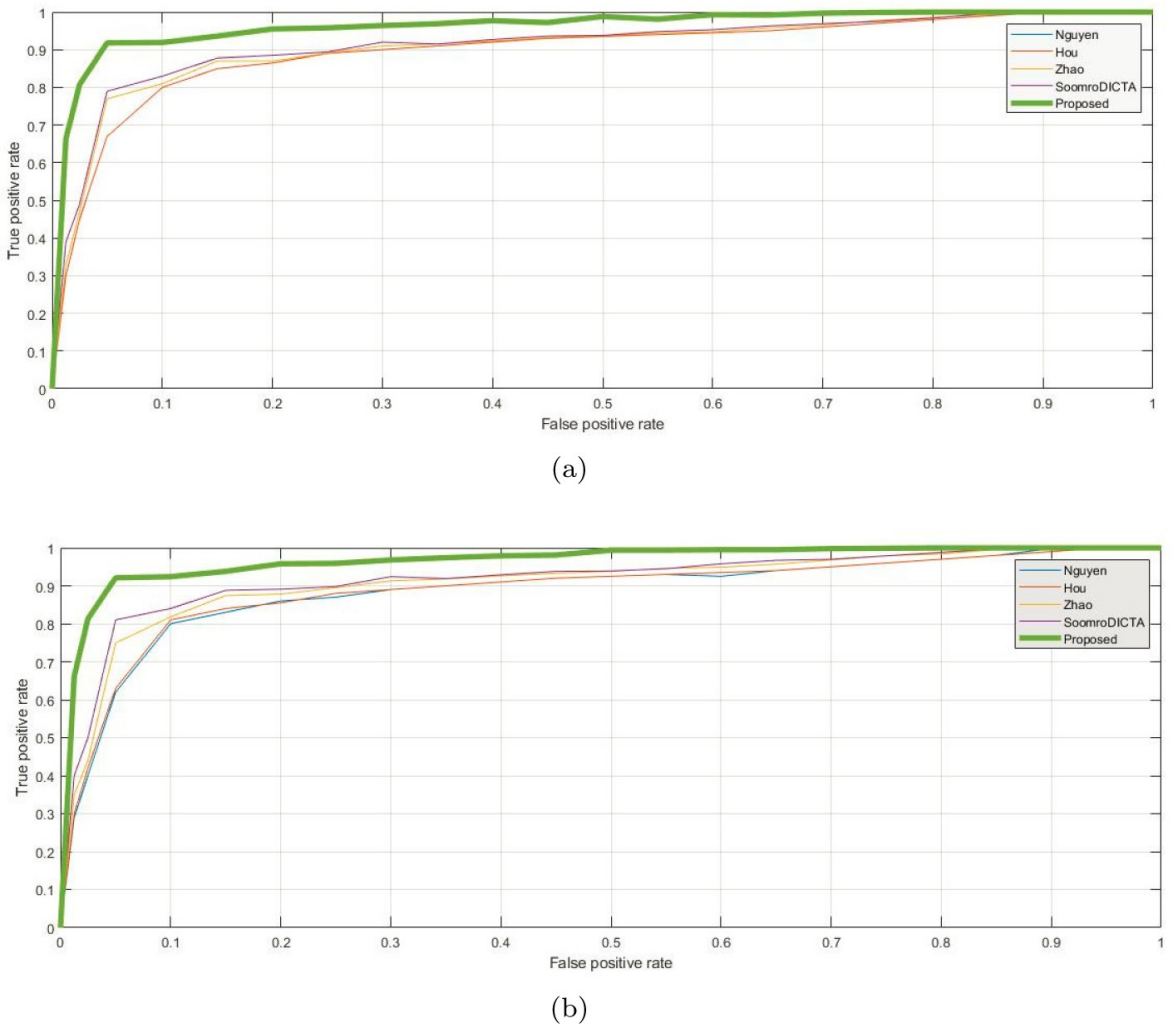
Receiver Operating Characteristic (ROC) curves for classification of vessels pixels on the DRIVE and STARE databases. In this Fig, the ROC curves indicate the performance of the proposed algorithm on the DRIVE (Fig(a)) and STARE (Fig(b)) databases.

We compared the performance of various methods for extracting retinal blood vessels from retinal colour fundus images using the Receiver Operating Curve (ROC) analysis as shown in the Fig 12. The ROC curve was computed by measuring the difference between the false positive rate and true positive rate of each method. The ROC curve is a graphical plot that illustrates the performance of a binary classifier system by plotting the true positive rate (sensitivity) against the false positive rate (1-specificity) at various decision thresholds. In other words, it shows the trade-off between sensitivity and specificity of a classifier at different levels of threshold values. A perfect classifier will have an ROC curve that passes through the upper left corner of the plot (100% sensitivity and 100% specificity). The closer the ROC curve is to the upper left corner, the better the performance of the classification of retinal vessels pixels. The results show (Fig 12) that the proposed method outperformed the existing methods, with a higher true positive rate and classification performance. However, the proposed method needs to be further validated on other databases, such as those for diabetic retinopathy, artery and vein occlusion, and hypertension retinopathy, to confirm its diagnostic accuracy. Nevertheless, the proposed method can provide an easy and accurate screening platform for ophthalmologists to detect retinal disorders.

### 6.4 Impact of 2nd order multi-dimensional LoG Detector on segmentation of retinal vessels

The aim of performing contrast scale normalization or 2nd order multi-dimensional LoG Detector is to detect vessel pixels, especially the tiny ones. In the proposed algorithm, the fivth stage involves a scale-normalization module, which depends on providing fixed values to the parameters *α* and *β*. It has been found that the performance of the proposed methods is related to the choice of optimal parameters. To determine the performance of the proposed method, empirical evidence was gathered by using four different scale-normalization cases, which are described as follows: case 1 with *α* = 0.5 and *β* = 0.5, case 2 with *α* = 1 and *β* = 0.5, case 3 with *α* = 1.5 and *β* = 0.5, and case 4 with *α* = 1 and *β* = 0.5. Table 3 shows the sensitivity, specificity, and accuracy data of the DRIVE and STARE databases for these four cases. The results obtained from the four different scale-normalisation cases show that accuracy increases with higher values of *α*, but sensitivity drops after *α* = 1. Since the objective of this study is to improve sensitivity, the optimal choice of parameters was determined to be case 2 with “*α* = 1 and *β* = 0.5”, as it achieves the highest sensitivity and accuracy.

**Table 3:** Impact of 2nd order multi-dimensional LoG Detector on segmentation of retinal vessels.

Databases	DRIVE			*STARE*		
Cases	*Se*	*Sp*	*AC*	*Se*	*Sp*	*AC*
Case 1:*α* = 0.5 and *β* = 0.5	0.751	0.951	0.912	0.742	0.949	0.902
Case 2: *α* = 1 and *β* = 0.5.	0.813	0.977	0.959	0.801	0.968	0.951
Case 3:*α* = 1.5 and *β* = 0.5.	0.798	0.962	0.935	0.786	0.958	0.931
Case 4 with *α* = 1 and *β* = 0.5.	0.801	0.967	0.943	0.799	0.961	0.948

### 6.5 Comparison with the enhancement methods

We perform a comparison of the results of our proposed technique with a new segmentation technique based on the enhancement technique, Yitian Zhao et al. [51]. Zaho et al. proposed methods based on enhancement filters and these filters are Frangi’s eigenvalue based filter (FR) [51, 52], Wavelet Filter (IUWT) [25, 51]. Table 4 shows the comparable performance of the technique that we proposed with other methods. It is evident that the method we developed produced far better results than these methods.

**Table 4:** Segmentation model performance assessment.

Database	DRIVE				*STARE*			
Measuring Parameters	*Se*	*Sp*	*AC*	*AUC*	*Se*	*Sp*	*AC*	*AUC*
Eigenvalue-based (FR) [51]	0.682	0.867	0.853	0.776	0.634	0.967	0.938	0.801
Wavelet (IUWT) [51]	0.716	0.978	0.9446	0.848	0.776	0.954	0.943	0.865
Proposed Method	0.813	0.977	0.959	0.895	0.801	0.968	0.951	0.885

**Fig 13:**
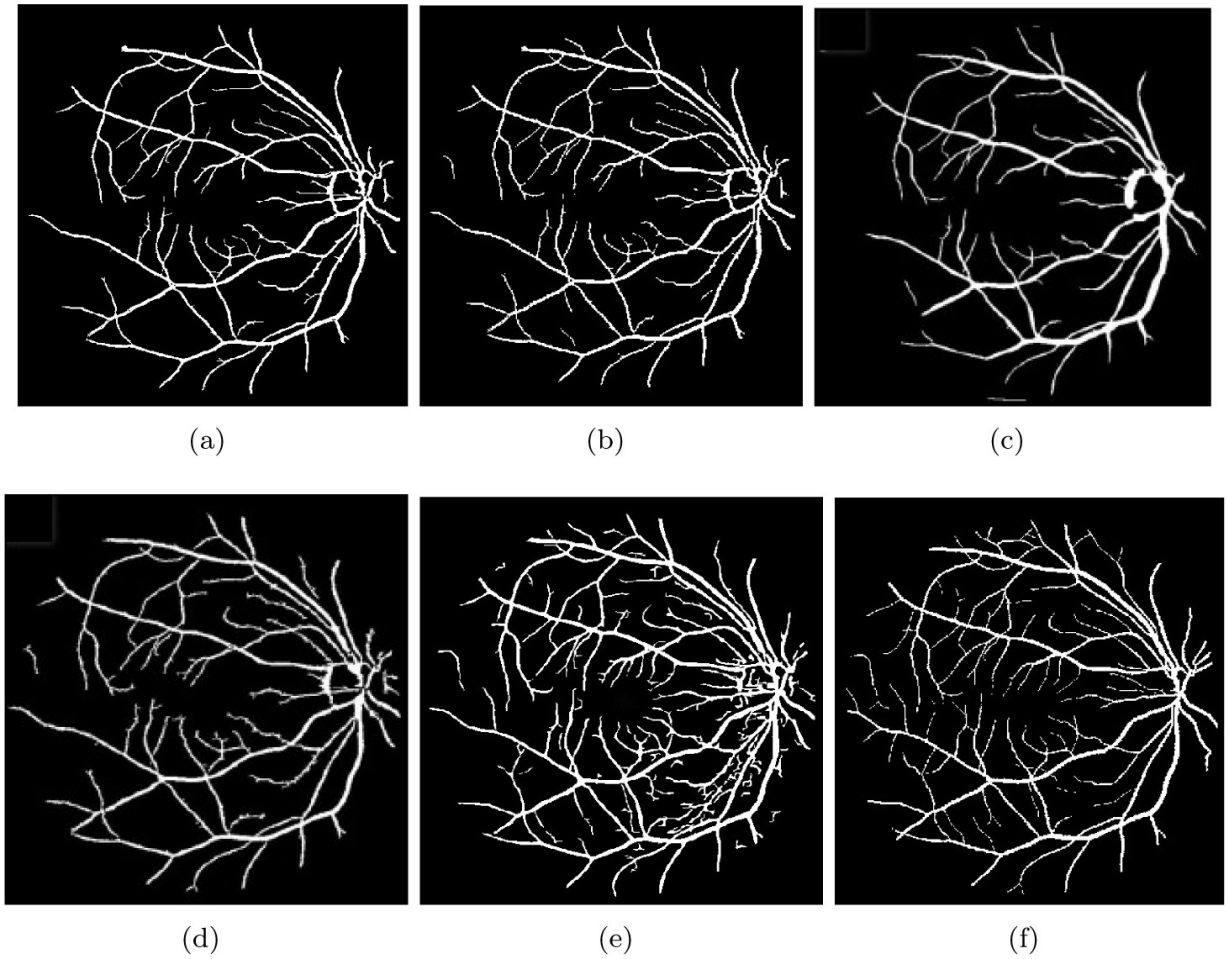
Comparative study of the results obtained by our proposed method and those of Nguyen et al. [49], Hou [50], and Zhao et al. [51] is presented. Fig (a) shows the result obtained by Nguyen, while Fig (b) shows the result obtained by Hou. Fig (c) and (d) show the results obtained by FR and IUWT-based Zhao, respectively. Fig (e) presents the results obtained by our proposed method, and Fig (h) shows the ground truth image.

The Fig 13 shows a comparison of vessel visualization between the proposed method and four other methods: Nguyen et al. [49], Hou [50], Frangi’s eigenvalue based filter (FR) [51, 52], and Wavelet Filter (IUWT) [25, 51]. It can be observed that the proposed method outperforms the other methods in detecting small blood vessels.

### 6.6 Comparative analysis

Our proposed technique for retinal vessel segmentation has undergone extensive validation against previously reported methods, using the STARE and DRIVE datasets. The evaluation results, summarized in Table 5, provide compelling evidence of the superiority of our approach. By comparing various performance metrics, we can gain a comprehensive understanding of its strengths and limitations.

**Table 5:** Comparison of proposed method with existing methods.

Database	DRIVE					STARE				
Methods	*Time*	*Se*	*Sp*	*AC*	*AUC*	*Time*	*Se*	*Sp*	*AC*	*AUC*
[19]	-	-	-	0.946	-	-	-	-	0.951	-
[54]	-	-	-	0.946	-	-	-	-	0.948	-
[55]	-	0.734	0.976	0.945	0.855	-	0.699	0.973	0.944	0.836
[56]	-	0.724	0.965	0.934	0.845	-	0.750	0.956	0.941	0.853
[26]	-	0.728	0.955	-	0.842	-	0.752	0.968	-	0.860
[57]	-	0.720	-	0.959	-	-	-	-	-	-
[58]	-	0.66	0.961	0.922	0.811	-	0.779	0.940	0.924	0.860
[20]	-	0.741	0.975	0.943	0.858	-	0.726	0.975	0.949	0.851
[33]	-	0.706	0.980	0.945	0.843	-	0.694	0.981	0.952	0.838
[59]	-	0.741	0.981	0.948	0.974	-	0.754	0.973	0.953	0.977
[49]	-	-	-	0.940	-	-	-	-	0.932	-
[50]	-	0.735	0.969	0.941	0.961	-	0.734	0.965	0.933	0.957
[60]	-	0.785	0.967	-	-	-	-	-	0.951	-
[61]	-	-	-	0.947	-	-	-	-	-	-
[62]	-	0.725	0.983	0.952	0.962	-	0.772	0.973	0.951	0.969
[63]	-	-	-	0.946	0.974	-	-	-	-	-
[64]	-	-	-	-	-	-	0.713	0.984	0.956	0.965
[65]	-	0.756	0.981	0.952	0.974	-	0.773	0.984	0.962	0.987
[51]	-	0.716	0.978	0.944	0.848	-	0.776	0.954	0.943	0.865
[66]	90s	0.713	0.968	0.941	0.841	91s	0.711	0.965	0.942	0.838
[48]	93s	0.734	0.967	0.951	0.850	95s	0.736	0.971	0.95	0.853
[67]	-	0.743	0.976	0.947	0.952	-	0.767	0.976	0.954	0.961
[68]	-	0.789	0.968	-	-	-	0.768	0.973	-	-
[69]	-	0.746	0.984	0.953	0.975	-	-	-	-	-
[70]	-	-	-	-	0.947	-	-	-	-	0.946
[31]	-	0.801	0.975	0.961	0.888	-	0.834	0.953	0.944	0.894
[71]	-	0.71	0.97	0.95	-	-	0.70	0.97	0.95	-
[72]	87s	0.752	0.976	0.953	-	89s	0.786	0.982	0.967	-
[37]	86s	0.745	0.962	0.948	-	89s	0.784	0.976	0.951	-
[53]	80s	0.812	0.971	0.963	0.951	81s	0.809	0.969	0.958	0.949
**Proposed Method**	**910ms**	**0.813**	**0.977**	**0.959**	**0.895**	**915ms**	**0.801**	**0.968**	**0.951**	**0.885**

In terms of sensitivity, our recommended approach excels, outperforming other methods in accurately detecting retinal vessels. However, it is important to highlight the findings of Thangaraj et al. [31], who achieved a higher sensitivity of 0.834 when using the STARE dataset. While their sensitivity surpasses our method in this specific aspect, it is crucial to consider the overall accuracy of the segmentation. Our approach, although slightly lower in sensitivity, compensates by delivering higher accuracy and improved performance in other areas.

On the other hand, Soomro et al. [53] achieved higher accuracy than our proposed technique, but at the expense of reduced sensitivity. This trade-off suggests that their method struggles to accurately detect small vessels, potentially limiting its effectiveness in certain applications that require precise delineation of fine retinal vasculature.

In addition to performance evaluation, we have also assessed the computational efficiency of our algorithm. By comparing the running time with those reported by other researchers (as indicated in Table 5), we found that our proposed method demonstrates faster execution while maintaining a commendable level of segmentation accuracy. This efficiency is particularly valuable in real-time applications or scenarios where processing time is a critical factor.

The results obtained through our proposed approaches validate their capability to accurately segment both small and large retinal blood vessels. This finding is significant, as it suggests that our method can effectively handle different vessel sizes, enabling its applicability in a wide range of retinal imaging tasks, such as disease diagnosis, treatment planning, and monitoring. Upon a thorough analysis of our proposed method’s performance, including rigorous experimental validation and comparison with state-of-the-art techniques, we have observed compelling evidence that highlights its exceptional capabilities. Our method demonstrates remarkable performance not only on standard databases but also on challenging images and pathological cases. It exhibits a superior ability to accurately detect even the tiniest retinal vessels, as substantiated by thorough quantification and visualization analyses.

In direct comparison to existing methods, whether they are machine learning-based or image processing-based, our proposed method consistently outperforms them. This superiority is evident across various performance metrics, establishing the effectiveness of our approach in achieving highly accurate retinal vessel segmentation. Moreover, it is worth noting that our method exhibits notable advantages in terms of computational efficiency. It demonstrates faster execution times compared to a multitude of existing methods, further enhancing its practicality and usability.

The comprehensive evaluation and analysis of our proposed method confirm its robustness and reliability, especially when confronted with challenging and pathological retinal images. Its ability to accurately detect tiny vessels sets it apart from other techniques, while its superior performance and efficient execution make it an ideal choice for real-world applications.

## 7 Discussion and future directions

Accurate segmentation of retinal blood vessels is a crucial step in developing an automated vessel analysis tool. Once the vessels are segmented accurately, it opens up opportunities for more advanced analysis, including measuring vessel diameters, assessing vessel tortuosity, distinguishing between veins and arteries, and ultimately aiding in disease diagnosis and grading. Although this paper focuses on evaluating the contrast-normalization steps specifically for retinal vessel segmentation, these steps can also be beneficial for analyzing larger retinal images. The proposed segmentation model demonstrates good performance in accurately segmenting vessels, even successfully detecting small vessels. Furthermore, the model’s flexibility makes it suitable for addressing segmentation challenges in medical images of other organs obtained through different imaging modalities such as CT, MRI, and X-ray. Introducing contrast normalization steps has the advantage of enhancing the robustness of the segmentation model, enabling it to effectively handle retinal images containing various pathologies.

However, it is important to acknowledge the limitations associated with the proposed method. Firstly, the evaluation of the segmentation model is limited to a specific set of retinal images from a particular database. This restricts the generalizability of the findings to other datasets or diverse populations. Additionally, the manual steps involved in the proposed method, such as managing the center of the light reflex and addressing low contrast variation, introduce subjectivity and potential inter-observer variability, affecting reproducibility and efficiency. Furthermore, while the proposed method achieves promising results compared to existing approaches, there is still room for improvement in terms of sensitivity and precision. Future research should focus on refining the method to enhance its performance. Additionally, the implementation of the proposed method into a practical software device for eye disease analysis is not detailed, and considerations regarding its integration into existing clinical workflows or software systems are not addressed.

Despite these limitations, the proposed method’s contribution to retinal vessel segmentation is significant, as it lays the foundation for more advanced vessel analysis and holds potential for application in medical images beyond retinal analysis. Further research and development are needed to address the limitations and validate the effectiveness and practicality of the proposed method in broader clinical settings.

## 8 Conclusion

An important procedure in diagnosing ocular abnormalities and assisting ophthalmologists is the analysis of retinal fundus images, particularly the enhancement or precise segmentation of retinal vessels. In this research, a novel method for retinal vessel segmentation is proposed. The initial steps of the method focus on addressing retinal image processing issues, managing the problem of the center of the light reflex, and handling low contrast variation using image processing techniques. The subsequent steps aim to achieve a well-segmented image by addressing the vessel consistency problem until a segmented vessel image is obtained. To evaluate the proposed method, it is tested on the DRIVE and STARE databases and compared with existing approaches. The evaluation shows that the proposed method achieves an average sensitivity of 0.81 and an average precision of 0.96, outperforming previous methods. However, there are still opportunities for improvement in the proposed method in the future. One potential avenue for improvement is training the retinal vessel methods using new deep learning models and incorporating a post-processing model to further enhance performance. This could lead to the development of a comprehensive software tool for eye disease analysis. Additionally, future enhancements may involve validating the performance of the model on larger databases containing diverse medical images.
